# Attenuation of estrogen and its receptors in the post-menopausal stage exacerbates dyslipidemia and leads to cognitive impairment

**DOI:** 10.1186/s13041-023-01068-0

**Published:** 2023-11-20

**Authors:** Qinghai Meng, Ying Chao, Shurui Zhang, Xue Ding, Han Feng, Chenyan Zhang, Bowen Liu, Weijie Zhu, Yu Li, Qichun Zhang, Huangjin Tong, Lixing Wu, Huimin Bian

**Affiliations:** 1 School of Medicine & Holistic Integrative Medicine, Nanjing University of Chinese Medicine, Nanjing, 210023 China; 2 School of Pharmacy, Nanjing University of Chinese Medicine, Nanjing, 210023 China; 3 Department of Pharmacy, Jiangsu Province Hospital of Integrated of Chinese and Western Medicine, Nanjing University of Chinese Medicine, Nanjing, 210028 China; 4 Department of Cardiovascular, Jiangsu Province Hospital of Integrated of Chinese and Western Medicine, Nanjing University of Chinese Medicine, Nanjing, 210028 China

**Keywords:** Menopause, Dyslipidemia, Cognition impairment, Estradiol, Estrogen receptor

## Abstract

Cognitive dysfunction increases as menopause progresses. We previously found that estrogen receptors (ERs) contribute to dyslipidemia, but the specific relationship between ERs, dyslipidemia and cognitive dysfunction remains poorly understood. In the present study, we analyzed sequencing data from female hippocampus and normal breast aspirate samples from normal and Alzheimer’s disease (AD) women, and the results suggest that abnormal ERs signaling is associated with dyslipidemia and cognitive dysfunction. We replicated a mouse model of dyslipidemia and postmenopausal status in LDLR^−/−^ mice and treated them with β-estradiol or simvastatin, and found that ovariectomy in LDLR^−/−^ mice led to an exacerbation of dyslipidemia and increased hippocampal apoptosis and cognitive impairment, which were associated with reduced estradiol levels and ERα, ERβ and GPER expression. In vitro, a lipid overload model of SH-SY-5Y cells was established and treated with inhibitors of ERs. β-estradiol or simvastatin effectively attenuated dyslipidemia-induced neuronal apoptosis via upregulation of ERs, whereas ERα, ERβ and GPER inhibitors together abolished the protective effect of simvastatin on lipid-induced neuronal apoptosis. We conclude that decreased estrogen and its receptor function in the postmenopausal stage promote neuronal damage and cognitive impairment by exacerbating dyslipidemia, and that estrogen supplementation or lipid lowering is an effective way to ameliorate hippocampal damage and cognitive dysfunction via upregulation of ERs.

**Graphical Abstract:**

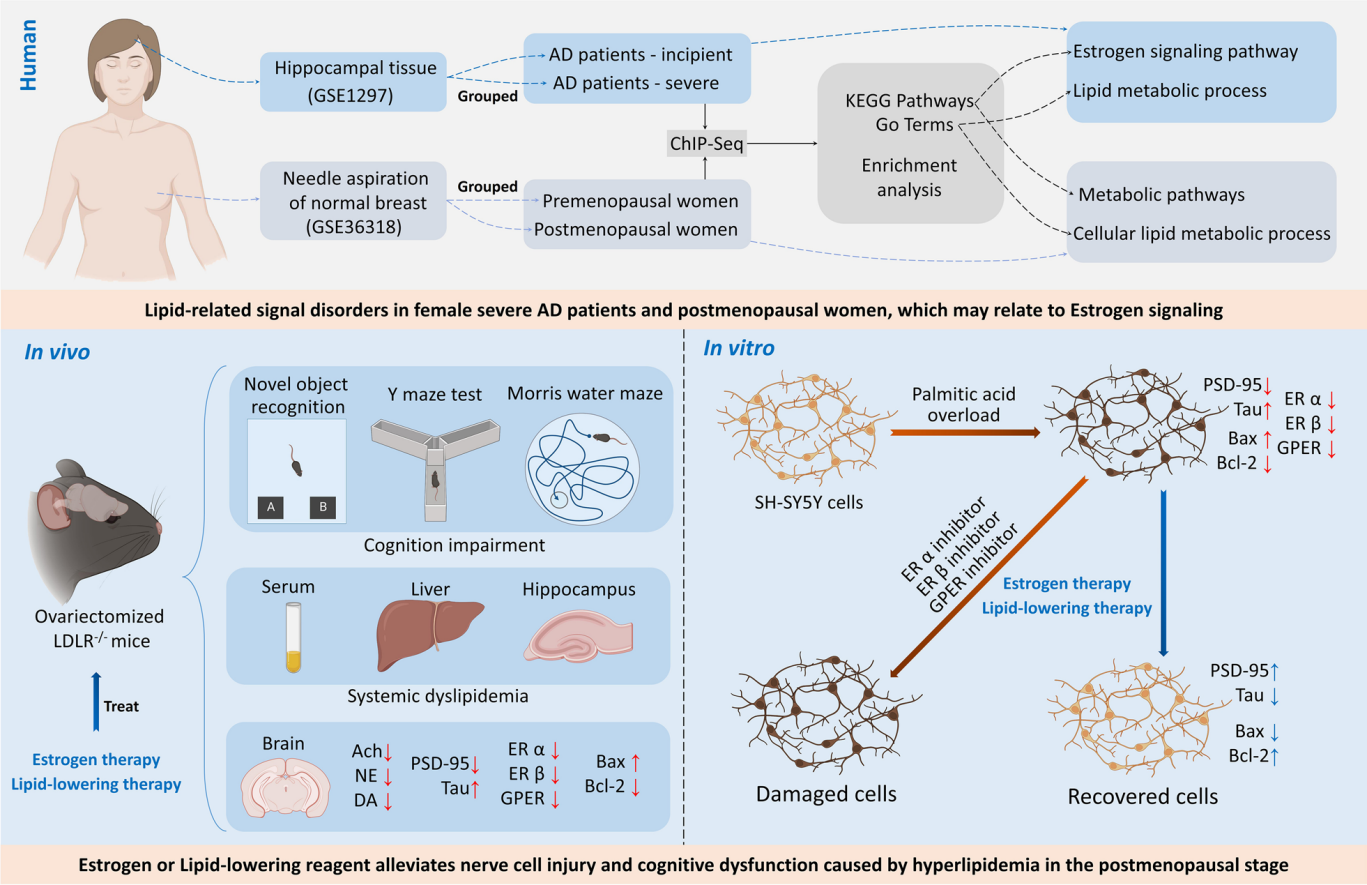

## Introduction

Menopause is a unique physiological transition period for women, during which the estrogen level in women decreases and the health status declines [1]. Changes in estrogen levels have a significant impact on the nervous system [2]. Cognitive abilities were assessed in premenopausal and postmenopausal women, and the results showed that premenopausal women had high estrogen levels and better cognitive abilities [3]. Compared with asymptomatic perimenopausal women and men, the Alzheimer’s disease (AD) indicators are increased in postmenopausal women with controlled age, including increased Aβ deposition and decreased gray matter and white matter volume in the AD-susceptible regions [4]. In a study, 72% of postmenopausal women claimed that they had memory problems [5]. The prevention and treatment of cognitive dysfunction has become a global health concern in postmenopausal women.

Changes in the endocrine system and metabolism occur in postmenopausal women, with a tendency towards hyperlipidemia and even metabolic syndrome [6]. Dyslipidemia has been shown to be an independent risk factor for diseases associated with cognitive impairment [7]. Dyslipidemia increases the apoptosis of hippocampal neurons by inducing inflammation and then leading to cognitive impairment-related behaviors [8]. Excessive lipid accumulation accelerates systemic inflammation and high levels of circulating free fatty acids, and the increase in free fatty acid content in the brain causes local inflammation and leads to decreased synaptic plasticity and neuronal damage in the hippocampus eventually [9]. A study has shown that statins, such as simvastatin, have therapeutic effects on cognitive impairment [10], so, regulating lipid levels to reduce hippocampal neuronal damage may be a way to improve cognitive function.

Ovariectomy in obese mice leads to increased metabolic disorders, hippocampal synaptic damage and cognitive dysfunction [11]. In the past, we have confirmed that blood lipid levels, especially TC and LDL-c levels, are significantly increased in ovariectomized mice [12], and we have found that the decrease in estradiol and its receptor function in ovariectomized mice is a key factor leading to dyslipidemia [13–15]. Estradiol plays a nutritional role in the development and function of the brain via ERs through the whole growth period [16]. A large number of studies have reported the physiological and pathological functions of estrogen and estrogen receptors (ERs) in the nervous system [2]. In diabetic mouse models, estrogen can inhibit nerve cell apoptosis and ameliorate cognitive dysfunction by inhibiting caspase-3 activity in the brain and increasing the bcl-2/Bax ratio [17]. In Aβ42-pretreated SH-SY5Y cells, estradiol increased cell viability and reduced cell apoptosis by inhibiting ROS production [18]. We hypothesized that the decline in estrogen and its receptors in the postmenopausal period may lead to cognitive impairment by exacerbating dyslipidemia. In the current study, we provide experimental evidence for this hypothesis, we showing that ER activation or lipid reduction may be alternative effective ways to reduce the occurrence of cognitive dysfunction in the postmenopausal stage.

## Materials and methods

### Regents

A total cholesterol assay kit (#A111-1-1), triglyceride assay kit (#A110-1-1), low-density lipoprotein cholesterol assay kit (#A113-1-1), high-density lipoprotein cholesterol assay kit (#A112-1-1), acetylcholine assay kit (#A105-1-1), choline acetyltransferase assay kit (#A079-1-1), acetylcholinesterase assay kit (#A024-1-1), dopamine assay kit (#H170), norepinephrine assay kit (#H096), and estradiol 2 assay kit (#H102-1) were purchased from Nanjing Jiancheng Bioengineering Institute (Nanjing, China). Alisol B 23-acetate (#PCS0958) was purchased from Chengdu Index Pure Biotechnology Co., Ltd. (Chendu, China). Estradiol (Lot: 356,564) was purchased from Abbott Healthcare Products (Netherlands). Simvastatin (Lot: 1,870,401) was purchased from Yangtze River Pharmaceutical Group (Taizhou, China). Palmitic acid (#SLBZ9610), MPP (#M70068), and PHTPP (#SML1355) were purchased from Sigma–Aldrich (New Jersey, USA). G-15 (#HY-103,449) was purchased from MedChemExpress (New Jersey, USA). PSD-95 antibody (#20665-1-AP), Tau antibody (#10274-1-AP), Bcl-2 antibody (#26593-1-AP), Bax antibody (#60267-1-Ig), β-actin antibody (#66009-1-Ig), and β-actin antibody (#20536-1-AP) were purchased from Proteintech (Wuhan, China). ERα antibody (#ab32063) was purchased from Abcam (Cambridge, England). ERβ antibody (#A2546) and GPER antibody (#A10217) were purchased from Abclone (Hangzhou, China). A metal enhanced DAB substrate kit (#DA1015), PMSF (100 mM) (#P0100), RIPA buffer (high) (#R0010), MTT cell proliferation and cytotoxicity assay kit (#M1020), and neutral balsam (#G8590) were purchased from Solarbio (Suzhou, China). Sensitive ECL chemiluminescent substrate (#BL520B), fetal bovine serum (#BL201A), high glucose DMEM (#BL301A), and trypsin solution (phenol red, EDTA-free) (#BL527A) were purchased from Biosharp (Hefei, China).

### GEO database analysis

The GSE36318 data set and GSE1297 data set were downloaded from the GEO database (https://www.ncbi.nlm.nih.gov/gds). Sample information and sequencing data were obtained by R studio. The groups were defined using GEO2R, and the sequencing data for the samples were standardized. The differentially expressed genes between the defined groups were analyzed by GEO2R, and a table of the results was downloaded and used for gene set enrichment analysis (https://david.ncifcrf.gov). The enrichment analysis results were visualized using a cloud plotting platform (http://vip.sangerbox.com).

### Experimental animal and postmenopausal dyslipidemia mouse model

C57BL/6J and LDLR^−/−^ mice (SPF grade, female, 15–20 g) were purchased from Jiangsu Jicuiyaokang Biotechnology Co., Ltd. All animals were raised in the Experimental Animal Center of Nanjing University of Chinese Medicine (Ethic No. 201912A012).

Before the experiment, the mice were adaptively fed an SPF grade experimental diet for 1 week. The groups included the C57BL/6J + normal diet group, C57BL/6J + high-fat diet group, LDLR^−/−^+ normal diet group, LDLR^−/−^+ high-fat diet group, LDLR^−/−^ ovariectomy + high-fat diet group, LDLR^−/−^ + ovariectomy + high-fat diet + 17-β estradiol (E2, 0.13 mg/kg) group and LDLR^−/−^ + ovariectomy + high-fat diet + simvastatin (SIM, 4.5 mg/kg) group. N = 5 in each group.

Mice accepting ovariectomy were anesthetized by intraperitoneal injection of 0.5% pentobarbital sodium. Then, a small incision was made on both sides of the back, and the ovaries were separated. After that, uterine ligation was performed, the ovaries were removed, and the wound was sutured on both sides of the back. One week after the operation, the mice were subjected to vaginal smears for 7 consecutive days, and the vaginal smears were observed under a microscope to observe whether the mice had an estrous cycle. The absence of the estrous cycle indicated that the mice were in a postmenopausal state and that the model was successfully replicated. Mice were given different diets according to the experimental groups. The administration group was treated once a day by gavage. The other groups were gastric administered 0.5% sodium carboxymethyl cellulose by gavage for 90 days.

### Cognitive behavior tests in mice

#### Novel object recognition test

Three objects A, B and C were used in the test; objects A and B were exactly the same, and object C was completely different from objects A and B. A box (0.4 m*0.4 m*0.3 m) was used to place the objects and mice. On the training day, objects A and B were placed on the two corners of one side of the box. The mice were then placed into the box with the back toward the two objects. Then, the video equipment was immediately opened to record the sniffing for the two objects of the mice for 5 min. After 24 h, object B was changed to object C, the mice were then placed into the box with the back toward the two objects again, and the sniffing of the two objects of the mice for 5 min was recorded. The discrimination index = (time to explore new things − time to explore old things)/ (time to explore new things + time to explore old things) *100%.

#### Y maze task

Before the experiment, the three arms of the Y maze were labeled arm A, arm B and arm C. The mice were placed in the same starting arm A with their heads facing the backplane. Then, the mouse was allowed to move freely for 8 min. A camera was used to record the behavior of the mice. Only when all four legs of the mouse entered one arm was it recorded as an arm entrance; when the mouse entered three different arms in turn, it was recorded as an alternate arm entrance. Maximum arm entrance = Total arm entrance − 2. Alternate behavior score = Total number of alternate arm entrances/maximum arm entrances * 100%.

#### Morris water maze

Positioning navigation experiments and space exploration experiments were included in the Morris water maze. The positioning navigation experiment was divided into a 4-day training and a one-day test. During the training period, mice were put into the pool from the entry point in each quadrant of the water maze and allowed to swim in the water and find the escape platform. The time limit for each quadrant was 60 s. During the test period, mice were put into each quadrant of the water maze to explore freely according to the training method, and the video software recorded the latency and swimming path of the mice. On the sixth day, a space exploration experiment was performed by removing the platform and putting the mice into the water facing the pool wall from the third quadrant to freely explore for 60 s. The video software recorded the times that cross the quadrant that the platform used to be placed.

### Detection of lipids in mice

Orbital blood was taken from all mice at the end of the administration. Centrifugation was performed at 3000 rpm/min for 10 min to collect the serum. TC, TG, HDL-C and LDL-C levels in serum were determined by referring to the instructions of the test kits. A small amount of hippocampal tissue or liver tissue was put into a precooled glass homogenate tube, and anhydrous ethanol was added at a ratio of 1:9 by mass volume and ground until the tissue was homogenized. Centrifugation was performed at 3000 rpm/min for 10 min, and the supernatant was collected. Then, the protein concentration of the sample was detected by using a BCA kit. Then, the TC and TG levels were detected by referring to the instructions of the test kits.

### ELISA and enzyme activity detection in mice

Orbital blood was taken from all mice at the end of the administration. Centrifugation was performed at 3000 rpm/min for 10 min before collecting the serum. The serum estradiol levels were determined by referring to the instructions of the estradiol ELISA detection kit. A small amount of hippocampal tissue was placed into a precooled glass homogenate tube, and saline or homogeneous solution provided by the kits was added at a ratio of 1:9 by mass volume and ground until the tissue was homogenized. Centrifugation was performed at 3000 rpm/min for 10 min, and the supernatant was collected. Then, the protein concentration of the sample was detected by using a NanoDrop System. The same total protein of the samples was used to detect Ach, NE, and DA levels according to the instructions of their ELISA detection kit. The activity of AChT and AchE in hippocampal tissues was detected by referring to the instructions of their detection kits.

### Mouse brain tissue staining

#### Nissl staining

The brain was fixed with 4% paraformaldehyde. After the brain was dehydrated, cleared and waxed, the wax blocks were obtained by paraffin embedding. A paraffin slicer was used for slicing, and sections with a thickness of 5 microns were obtained. The sections were dewaxed with xylene and then rehydrated, soaked and stained with Nissl’s dye A solution according to a Nissl staining kit. After differentiation with Nissl’s dye B solution, the sections were stained with hematoxylin. After that, the sections were dehydrated, cleared and sealed. A microscope was used to observe and take photographs of the brain.

#### Immunohistochemistry staining

Brain sections of 5 microns thickness were obtained. After the sections were dewaxed, rehydrated, and soaked in 3% hydrogen peroxide, they were washed with PBS 3 times. Then, the sections were soaked in citrate buffer at 95 °C for 20 min. After cooling, the slides were sealed with 5% BSA for 20 min. Primary antibodies against ERα, ERβ, and GPER were incubated with the brain overnight at 4 °C. After washing with PBS 3 times, the brain was incubated with the secondary antibody for 2 h at room temperature. A DAB chromogenic kit was used to incubate the brain for color development. After washing with PBS 3 times, the sections were stained with hematoxylin. After that, the sections were dehydrated, cleared and sealed. A microscope was used to observe and take photographs of the brain.

#### Immunofluorescence staining

Brain sections of 5 microns thickness were prepared. Following dewaxing and rehydration, the sections were treated with 3% hydrogen peroxide, then washed with PBS three times. Subsequently, the sections were immersed in citrate buffer at 95 °C for 20 min. After cooling, the slides were blocked with 5% BSA for 20 min. Primary antibodies against PSD-95 and TAU were applied to the sections and incubated overnight at 4 °C. After washing with PBS three times, the sections were incubated with the fluorescently-labeled secondary antibody for 2 h at room temperature. The sections were then covered with an anti-fade mounting medium containing DAPI. Photographs were taken using a fluorescence microscope.

#### TUNEL staining

Brain sections of 5 microns thickness were processed. After dewaxing, rehydrating, and treating with 3% hydrogen peroxide, the sections were washed three times with PBS. The sections were then immersed in citrate buffer at 95 °C for 20 min before cooling. The TUNEL reaction mixture was then applied to the sections according to the manufacturer’s instructions and incubated in a humidified atmosphere. Following this, the sections were washed thoroughly with PBS. They were then incubated with a FITC-conjugated secondary antibody specific to the TUNEL reaction. To counterstain the nuclei, the sections were covered with an anti-fade mounting medium containing DAPI. Observations and photographs of the fluorescently-labeled apoptotic cells were taken using a fluorescence microscope.

### Western blot of the brains of mice

The hippocampus of the mice was collected, and the lysate was prepared according to the ratio of RIPA:PMSF = 100:1. The lysate was added according to the ratio of weight: volume = 50 mg: 1 mL. Two steel balls with a diameter of 3 mm were added, the tissue homogenizer was set at 50 Hz, and the brains were crushed for 30 s. Centrifugation was performed at 12,000 rpm/min for 10 min to collect the supernatant. The protein concentration of the sample was detected by using a NanoDrop System. Then, 5× sample loading buffer was added and cooked at 100 °C for 15 min on a dry thermostat. After SDS–PAGE and membrane transfer, primary antibodies against ERα (55 kDa), ERβ (65 kDa), GPER (55 kDa), PSD-95 (80 kDa), Tau (79 kDa), Bax (21 kDa), and Bcl-2 (26 kDa) were diluted according to the manufacturer’s instructions. As for loading control, a separate piece of membrane loaded with the same amount of protein of the samples was incubated with β-actin (43 kDa). The PVDF membrane was then incubated overnight with the diluted primary antibodies in a refrigerator at 4 °C. The membrane was washed the next day, and the second antibody was used to incubate the membrane at room temperature for 2 h. After washing again, a chemiluminescence kit and gel imaging system were used to expose the blots and analyze the data using relatively quantitative methods.

### Culture and intervention of SH-SY5Y cells

SH-SY5Y cells were cultured with DMEM complete medium with 10% FBS in a 25T cell flask in an incubator at 37 °C and 5% CO_2_. When the density of SH-SY5Y cells reached 80%, conventional subculture was performed. Palmitic acid (PA, 0.0307 g) was added to 3 mL of sodium hydroxide solution (0.1 mM) to prepare the PA storage solution. The mixture was placed at 75 °C for 30 min. A 40% BSA solution was mixed with the PA storage solution in a 1:1 ratio to obtain a 20 nM PA working solution. The PA working solution was then diluted to different concentrations as required and used for intervention in SH-SY5Y cells. AB23A (40 µM), MPP (10 µM), PHTPP (10 µM), and G15 (100 nM) were also used to treat SH-SY5Y cells according to different requirements.

### Cell viability detection of SH-SY5Y cells

When the density of SH-SY5Y cells reached 80%, the medium was discarded, and 2 mL trypsin solution was added to collect the cells. Then, SH-SY5Y cells were counted by the cell counter and diluted with medium to seed into a 96-well plate with 1 × 10^4^ cells per well. The cells were cultured in the incubator until the density of SH-SY5Y cells reached 80%. Different concentrations of PA were added as needed. After incubation, 200 µL MTT solution was added to each well. The 96-well plate was placed in a 37 °C incubator and incubated for 4 h. Finally, the supernatant was discarded, and 200 µL DMSO was added to each well. The absorbance (OD) value was measured at 490 nm by using a microplate reader.

### Immunofluorescence staining of SH-SY5Y cells

SH-SY5Y cells were grown on 24-well plates. Before cell seeding, a circular sterile glass sheet of 24 mm was added to each well. Cells were then cultured and treated with different reagents. After a specific time, the culture medium was discarded, and the wells were washed twice with PBS. Then, SH-SY5Y cells were fixed with 4% paraformaldehyde and permeabilized with 0.2% Triton for 20 min. SH-SY5Y cells were incubated with 5% BSA for 20 min. Primary antibodies against ERα, ERβ, GPER, PSD-95 and Tau, according to the instructions, were diluted and added to incubate the cells overnight at 4 °C. On the second day, after washing with PBS three times, the secondary antibody was added, and the cells were incubated at room temperature for 2 h. After washing with PBS three times, the cells were incubated with DAPI for 5 min to stain the nucleus. After washing with PBS three times, the sterile glass sheets were removed and sealed with glycerin-gelatin sealing liquid. A fluorescence microscope was used to observe and take photographs of the SH-SY5Y cells.

### Western blot of SH-SY5Y cells

The medium was discarded from the petri dish, and the SH-SY5Y cells were rinsed with 500 µL PBS solution. Then, 250 µL of mixed lysate was added to each dish, and the SH-SY5Y cells were lysed for 0.5 h. Centrifugation was performed at 12,000 rpm/min for 10 min to collect the supernatant. The protein concentration of the samples was detected by using a Nano Drop System.

The following operations were the same as “Western blot of brain in mice”.

### Statistical analysis

The sample information of the GSE36318 data set and GSE1297 data set was compared by unpaired parametric t test. ImageJ was used to count Nissl bodies in the hippocampus of mice and exported measurement data. Image-Pro Plus 6.0 was used to quantify the mean optical density of immunostaining in in vivo and in vitro experiments and exported measurement data. Quantity One 4.5.2 was used to perform the relative quantification of protein expression in in vivo and in vitro experiments and exported measurement data. All measurement data are shown as the mean ± standard deviation. An unpaired parametric t test was used to analyze the difference between two groups. Ordinary one-way ANOVA with multiple comparisons was used to analyze the difference between more than two groups. When *P* < 0.05, the difference was considered significant. All measurement data were visualized and analyzed using GraphPad 9.0.

## Results

### Lipid-related signal disorders in postmenopausal women and in women with cognitive impairment

**Fig. 1:**
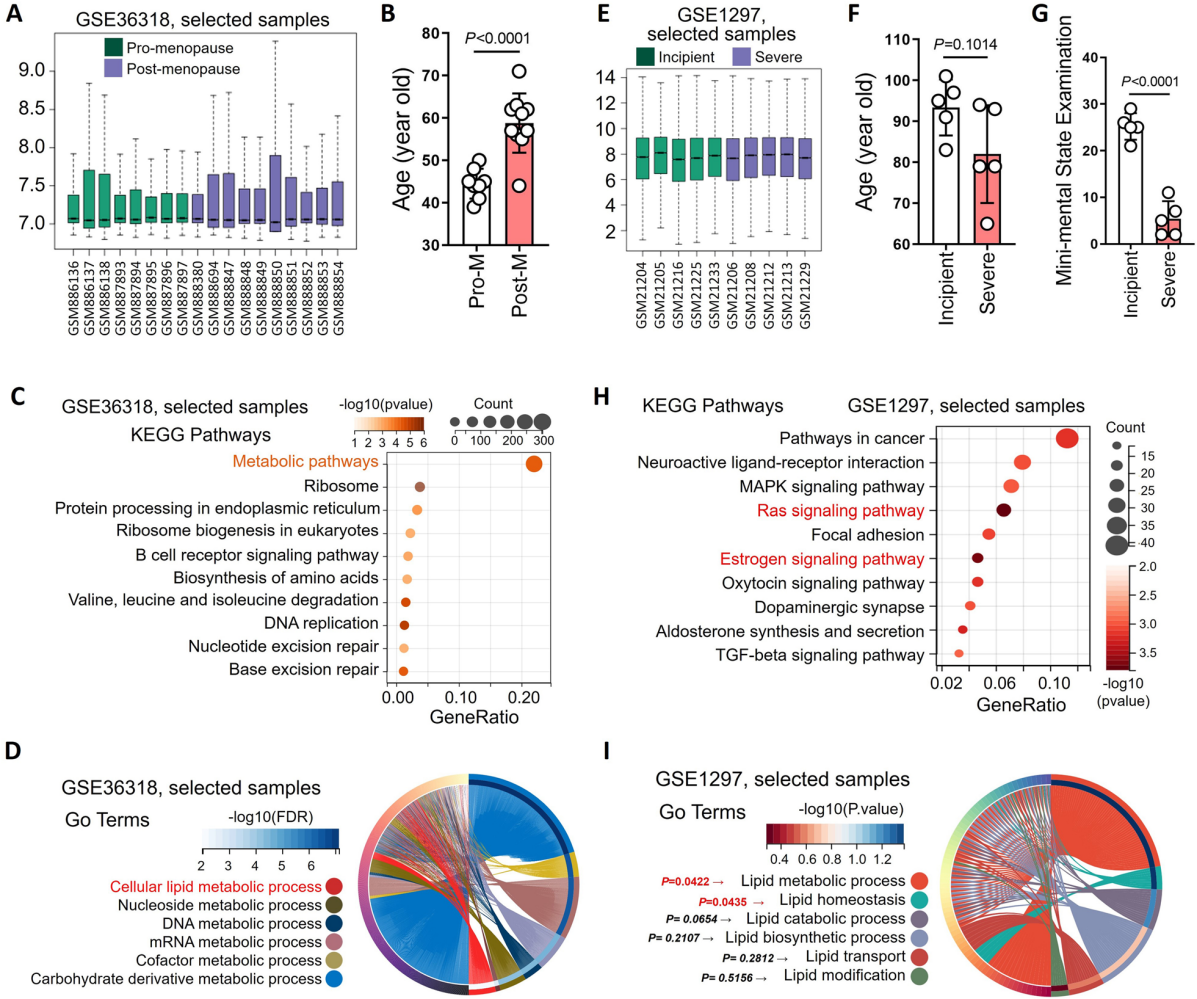
Lipid-related signal disorders in postmenopausal women and in women with cognitive impairment. The sample information and the sequencing results of random needle aspiration samples from normal breast tissue of premenopausal (n = 8) and postmenopausal (n = 10) women were extracted and analyzed from the GSE36318 data set. **A** The groups were defined using GEO2R, and the sequencing data for the samples were standardized. **B** Ages were extracted from sample information, and histograms were drawn to analyze differences between premenopausal (n = 8) and postmenopausal (n = 10) women. **C** GEO2R was used to analyze genes that were differentially expressed between premenopausal and postmenopausal women. KEGG signal enrichment analysis was performed with differentially expressed genes between the two groups, and a bubble diagram was drawn. **D** GO-MP signal enrichment analysis was performed with differentially expressed genes between the premenopausal and postmenopausal women, and a loop graph was drawn. The sample information and the sequencing results of hippocampal tissue from female incipient AD patients (n = 5) and severe AD (n = 5) patients were extracted and analyzed from the GSE1297 data set. **E** The groups were defined using GEO2R, and the sequencing data for the samples were standardized. **F** Ages were extracted from sample information, and histograms were drawn to analyze differences between female incipient AD patients and severe AD patients. **G** Mini-mental state examination results were extracted from sample information, and a histogram was drawn to analyze the differences between female incipient AD patients and severe AD patients. **H** GEO2R was used to analyze genes that were differentially expressed between female incipient AD patients and severe AD patients. KEGG signal enrichment analysis was performed with differentially expressed genes between the two groups, and a bubble diagram was drawn. **I** GO-MP signal enrichment analysis was performed with differentially expressed genes between the female incipient AD patients and severe AD patients, and a loop graph was drawn

Sequencing data of random needle aspiration samples from normal breast tissue of premenopausal and postmenopausal women were analyzed using the GSE36318 data set from the GEO database (Fig. 1A). Sample information showed that postmenopausal women were significantly older than premenopausal women (Fig. 1B). KEGG enrichment analysis results showed that metabolic pathways were the most significantly altered among the top ten significantly changed signaling pathways (Fig. 1C). Biological process (BP) of gene ontology (GO) results showed that differentially expressed genes were enriched in various material metabolic signals, including cellular lipid, nucleoside, DNA, mRNA and cofactor metabolic processes. More than 40% of the differentially expressed genes were enriched in cellular lipid metabolic processes (Fig. 1D).

Sequencing data of hippocampal tissue of female AD patients were analyzed using the GSE1297 data set from the GEO database (Fig. 1E). Sample information showed that there was no significant difference in age between the incipient and severe groups (Fig. 1F). The mean point of mini-mental state examination of the incipient group was close to the threshold for dementia diagnosis (23 points), while it was close to 5 in the severe group (Fig. 1G). KEGG enrichment analysis of differentially expressed genes in the hippocampus of incipient AD and severe AD women indicated the top ten significantly changed signaling pathways. Among them, the Ras signaling pathway and estrogen signaling pathway had the most significant changes in p value, and pathways in cancer had the most enriched genes (Fig. 1H). Furthermore, the BP GO enrichment analysis of differentially expressed showed that the lipid-related processes showed significant changes, including lipid metabolic processes, lipid homeostasis, lipid catabolic processes, lipid biosynthetic processes, lipid transport, and lipid modification (Fig. 1I).

### Ovariectomy leads to systemic dyslipidemia and cognitive impairment in LDLR^−/−^ mice

**Fig. 2:**
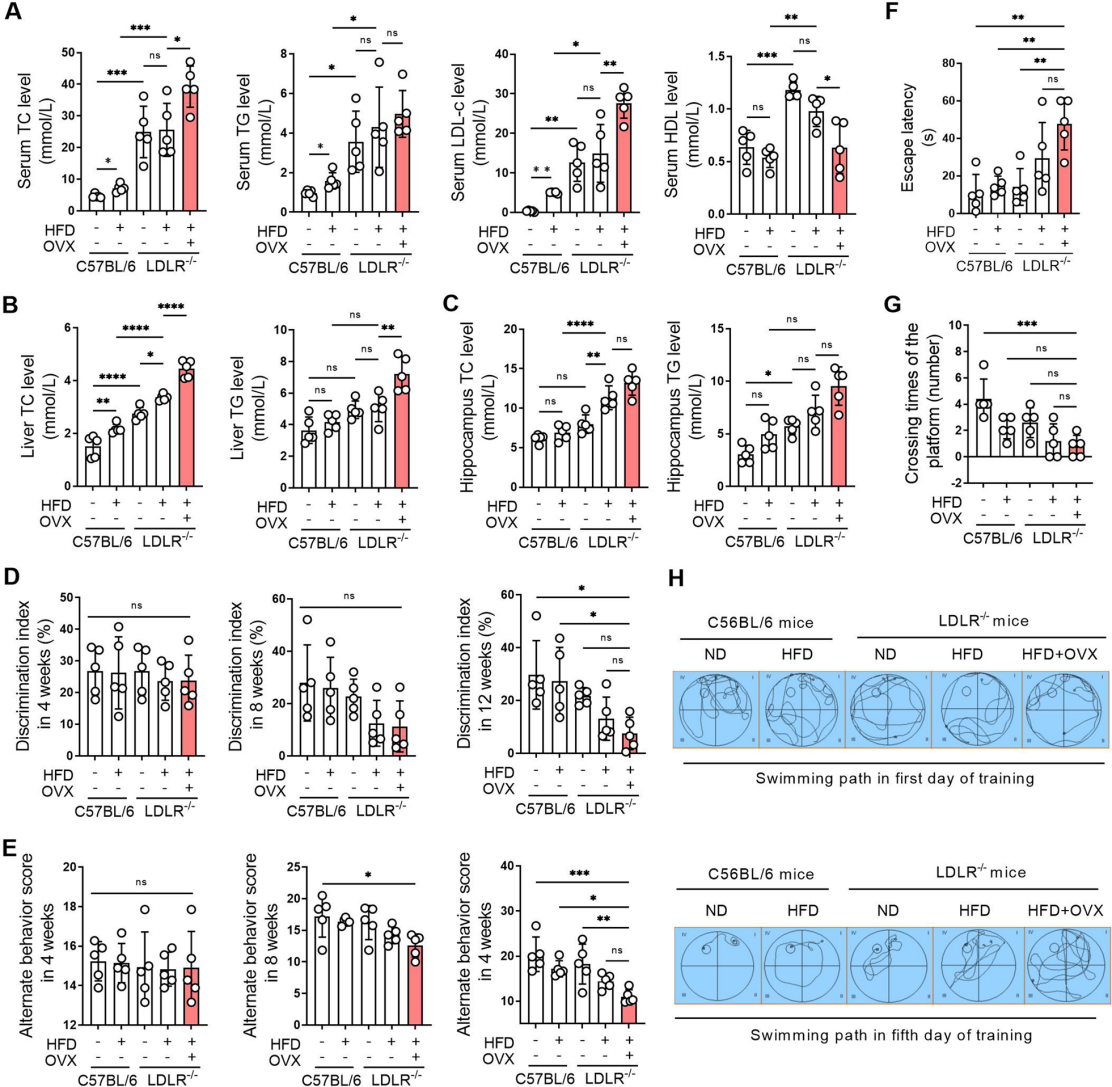
Ovariectomy leads to systemic dyslipidemia and cognitive impairment in LDLR ^−/−^ mice. C57BL/6 and LDLR^−/−^ mice were fed a normal diet (ND) or a high-fat diet (HFD) for 90 days. Bilateral ovariectomy was performed in LDLR^−/−^ mice fed a HFD to simulate a postmenopausal stage. **A** Serum of mice was obtained after 90 days of administration, and the levels of TC, TG, LDL-c and HDL-c were detected using biochemical kits. **B** Livers of mice were obtained after 90 days of administration, and the levels of TC and TG in liver homogenates were detected using biochemical kits. **C** The hippocampi of mice were obtained after 90 days of administration, and the levels of TC and TG in hippocampal homogenates were detected using biochemical kits. **D** The learning and memory ability of mice was evaluated by the novel object recognition test after 4, 8, and 90 days of administration. **E** Spatial memory of mice was evaluated by the Y-maze task after 4, 8, and 90 days of administration. **F**–**H** The learning and memory abilities of mice were evaluated by the Morris water maze after 4, 8, and 90 days of administration. **F** indicates the escape latency of mice on the fifth training day of the Morris water maze test. **G** Indicates the number of platform crossings on the sixth day of the Morris water maze test. **H** Shows the swimming paths of mice on the first and fifth training days of the Morris water maze test. N = 5. In the indicated comparison, *P < 0.05, **P < 0.01, ***P < 0.001, ****P < 0.0001

Compared with C57BL/6J mice fed a normal diet, the levels of serum TC, TG, LDL-c and HDL-c were significantly increased in LDLR^−/−^ mice fed a normal diet. Compared with LDLR^−/−^ mice fed a normal diet, there were no significant differences in serum TC, TG, LDL-c and HDL-c levels in LDLR^−/−^ mice fed a high-fat diet. Compared with LDLR^−/−^ mice fed a high-fat diet, TC and LDL-c levels were significantly increased in LDLR^−/−^ ovariectomized mice fed a high-fat diet, suggesting that ovariectomy in LDLR^−/−^ mice exacerbated hyperlipidemia (Fig. 2A). The levels of TC and TG in LDLR^−/−^ mice fed a normal diet were significantly increased compared to C57BL/6J mice fed a normal diet in the liver and hippocampus. The TC and TG levels in the liver and hippocampus were further increased by ovariectomy in LDLR^−/−^ mice (Fig. 2B, C).

New object recognition test results showed that there was no significant difference in the discrimination index of each group of mice after 4 weeks administration. After 8 weeks administration, the discrimination index showed a decreasing trend in LDLR^−/−^ mice fed a high-fat diet and in ovariectomized LDLR^−/−^ mice fed a high-fat diet. After 90 days administration, ovariectomized LDLR^−/−^ mice fed a high-fat diet showed the lowest discrimination index in all groups, indicating that the learning and memory ability of the mice had been severely impaired by ovariectomy (Fig. 2D). The Y maze task showed that there was no significant difference in the alternate behavior score of each group of mice after 4 weeks of administration. After 8 weeks administration, the alternate behavior score showed a decreasing trend in ovariectomized LDLR^−/−^ mice fed a high-fat diet. After 90 days administration, the alternate behavior score was significantly decreased in ovariectomized LDLR^−/−^ mice fed a high-fat diet compared to other groups, indicating that the spatial memory of the mice had been severely impaired by ovariectomy (Fig. 2E). The Morris water maze showed that after 90 days administration, the escape latency of ovariectomized LDLR^−/−^ mice fed a high-fat diet was significantly longer and the number of platform crossings was reduced compared to other groups. On the fifth day, the swimming path of ovariectomized LDLR^−/−^ mice fed a high-fat diet was the most complicated among all groups, reflecting confusion in the exploration and impaired learning and memory abilities of the ovariectomized mice (Fig. 2F–H).

### Ovariectomized LDLR^−/−^ mice showed severe neurotransmitter abnormalities and hippocampal damage

**Fig. 3:**
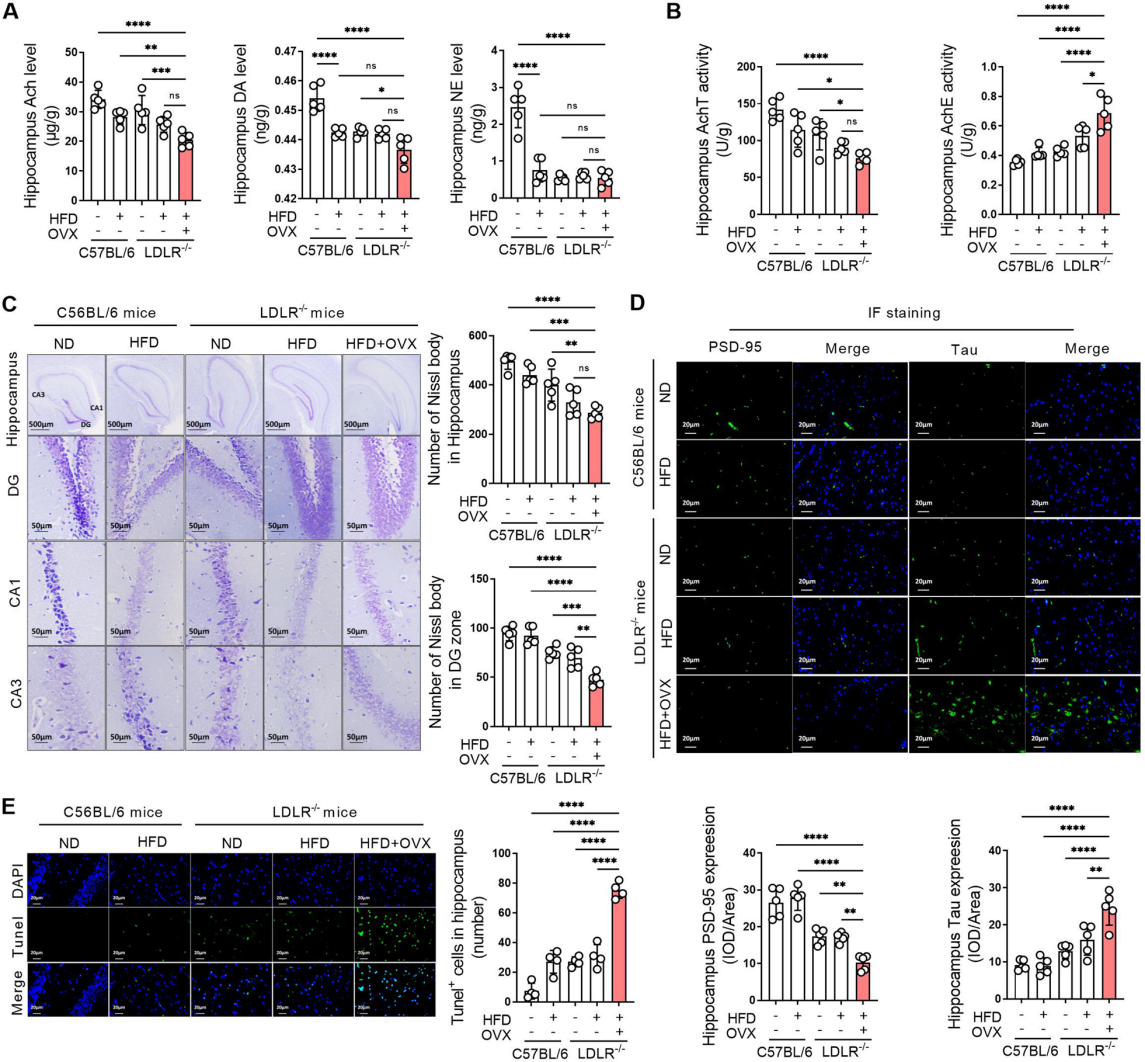
Ovariectomized LDLR ^−/−^ mice showed severe neurotransmitter abnormalities and hippocampal damage. C57BL/6 and LDLR^−/−^ mice were fed a ND or HFD for 90 days. Bilateral ovariectomy was performed in LDLR^−/−^ mice fed a HFD to simulate a postmenopausal stage. **A** The hippocampi of mice were obtained after 90 days of administration, and the levels of Ach, DA and NE in hippocampal homogenates were detected using ELISA kits. **B** The activities of AChT and AchE in hippocampal homogenates of mice were detected using biochemical kits after 90 days of administration. **C** The hippocampi of mice were obtained after 90 days of administration. The changes in Nissl bodies in hippocampal tissues were detected by Nissl staining. The representative images show the changes in Nissl bodies in the whole hippocampus, DG, CA1 and CA3 areas, and the magnification and scale bar are marked in the representative images. The quantity of Nissl bodies in hippocampal tissue and the DG region was quantified. **D** The expression of PSD-95 and Tau in the hippocampal tissues of mice was detected by immunofluorescence staining after 90 days of administration. The magnification and scale bar are marked in the representative images. The expression levels of PSD-95 and Tau in the hippocampus were quantified by the mean optical density. **E** The cell apoptosis in the hippocampal tissues of mice was detected by tunel staining after 90 days of administration. The number of apoptotic cells in each view was counted. **A**–**D**, n = 5. **E**, n = 4. In the indicated comparison, *P < 0.05, **P < 0.01, ***P < 0.001, ****P < 0.0001

Compared with C57BL/6J mice fed a normal diet, norepinephrine (NE) and dopamine (DA) levels in other groups were significantly decreased. Ovariectomy led to a decrease tendency in acetylcholine (Ach) levels in LDLR^−/−^ mice fed a high-fat diet (Fig. 3A). Compared with C57BL/6J mice fed a normal diet, hippocampal AChE activity increased and AChT activity decreased in LDLR^−/−^ mice fed a normal diet. The changes in AchTE and AChE activity were exacerbated by the high-fat diet in LDLR^−/−^ mice, and AChT activity further decreased and AchE activity further increased in LDLR^−/−^ mice fed a high-fat diet when ovariectomy was performed (Fig. 3B). Nissl bodies in the CA1, CA3, and DG areas of the hippocampus of C57BL/6J mice fed a normal diet were arranged neatly and closely with a liberal quantity. The hippocampus of the LDLR^−/−^ mice fed a high-fat diet showed fewer and scattered arrangement Nissl bodies. The number of Nissl bodies in the hippocampus was further reduced after ovariectomy performed in LDLR^−/−^ mice fed a high-fat diet (Fig. 3C). Compared with C57BL/6J mice, PSD-95 expression was decreased and Tau expression was increased noteworthy in LDLR^−/−^ mice. After ovariectomy, PSD-95 expression in the hippocampus was significantly decreased, and Tau expression was significantly increased compared to that in the other groups (Fig. 3D). Compared to the C57BL/6J mice fed a normal diet, the hippocampal regions of LDLR^−/−^ mice displayed an increase number of TUNEL-positive cells, indicating enhanced cell apoptosis. This trend was even more pronounced in LDLR^−/−^ mice fed a high-fat diet post-ovariectomy, further supporting the detrimental effects of hormonal changes on neuronal health. The quantification of apoptotic cells in each view highlighted these significant differences among the groups (Fig. 3E).

### ERs remarkably decrease and positively correlate with hyperlipidemia and cognitive impairment in ovariectomized LDLR^−/−^ mice

**Fig. 4:**
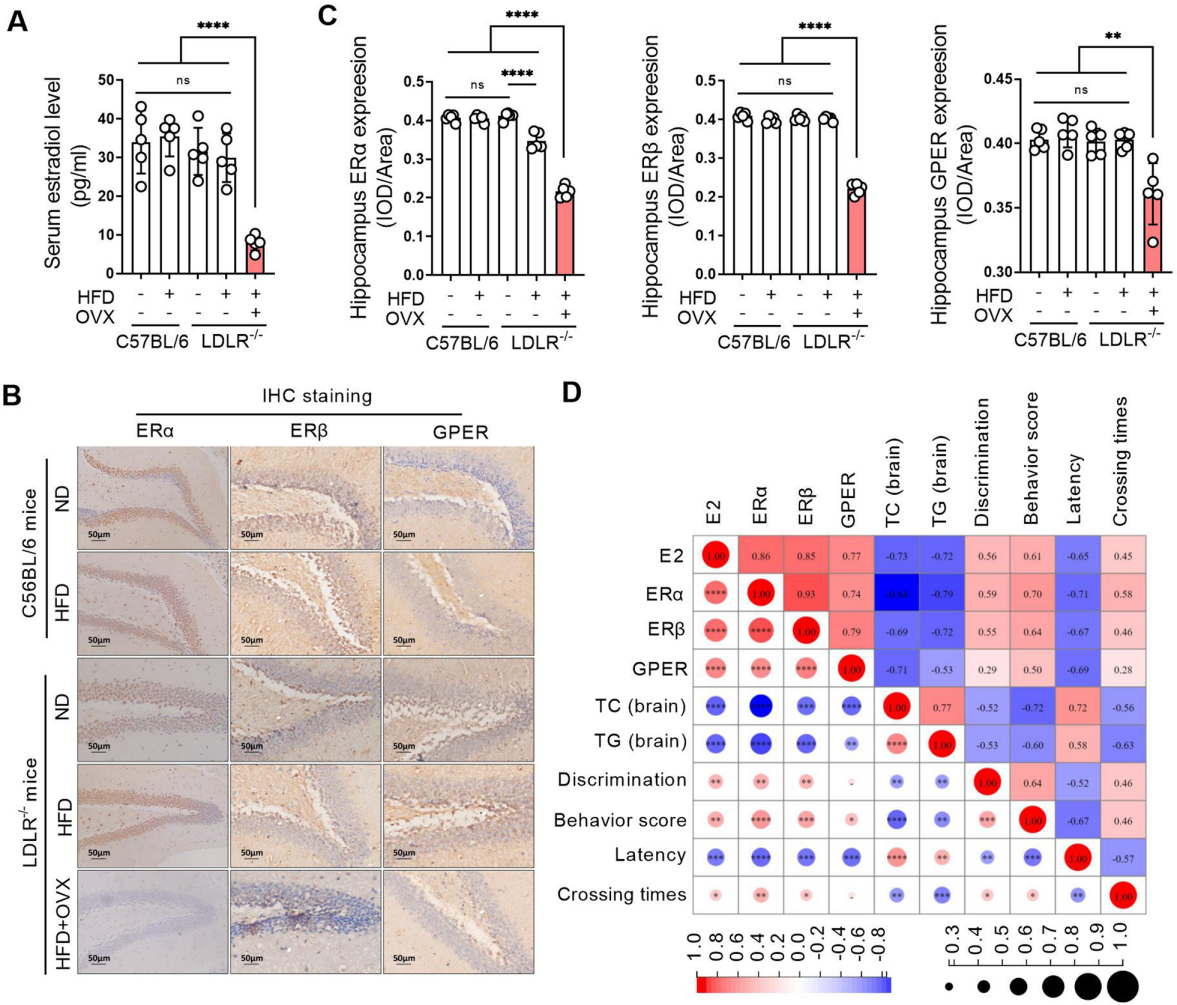
ERs remarkably decrease and positively correlate with hyperlipidemia and cognitive impairment in ovariectomized LDLR ^−/−^ mice. C57BL/6 and LDLR^−/−^ mice were fed a ND or HFD for 90 days. Bilateral ovariectomy was performed in LDLR^−/−^ mice fed a HFD to simulate a postmenopausal stage. **A** Serum of mice was obtained after 90 days of administration, and the serum estradiol levels were detected using an ELISA kit. **B** The expression of ERα, ERβ and GPER in the hippocampal tissues of mice was detected by immunohistochemistry staining after 90 days of administration. The magnification and scale bar are marked in the representative images. **C** Related to **B**, the expression levels of ERα, ERβ and GPER in the hippocampus were quantified by mean optical density. **D** Pearson was used to conduct correlation analysis on serum E2 levels, hippocampal ERα, ERβ and GPER expression, hippocampal TC and TG levels, and cognitive behavior test results. The red background indicates a positive correlation, and the blue background indicates a negative correlation. The number in the circle indicates the correlation coefficient, and the symbol * in the circle indicates the significance. N = 5. In the indicated comparison, *P < 0.05, **P < 0.01, ***P < 0.001, ****P < 0.0001

There was no difference in serum estradiol levels in mice without ovariectomy, indicating that neither LDLR knockout nor a high-fat diet had any effect on estradiol levels in mice. Estradiol levels in ovariectomized mice were significantly lower than those in the other groups (Fig. 4A). The expression of ERα, ERβ and GPER did not change significantly in the hippocampal tissues in mice without ovariectomy, while the expression of ERα, ERβ and GPER in hippocampal tissues of ovariectomized mice was significantly decreased compared with that of no ovariectomized mice (Fig. 4B, C). Correlation analysis showed that serum estrogen and ERα, ERβ and GPER expression in the hippocampus were negatively correlated with TC and TG levels in the hippocampus of mice, but they positively correlated with cognitive behavior scores of mice, and the TC and TG levels in the hippocampus of mice were negatively correlated with the cognitive behavior score of mice (Fig. 4D).

### Lipid overload leads to decreased ERs and promotes apoptosis in SH-SY5Y cells

**Fig. 5:**
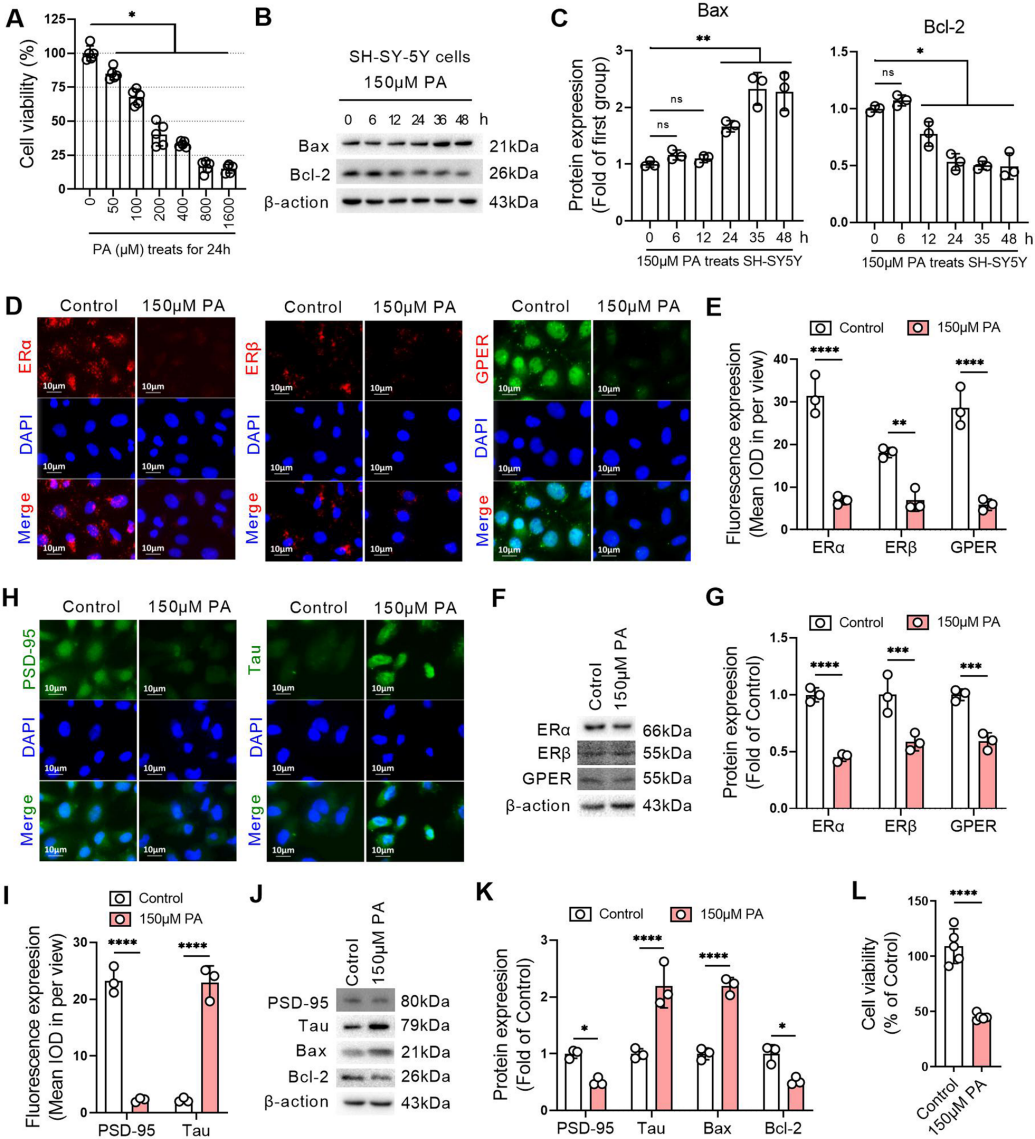
Lipid overload leads to decreased ERs and promotes apoptosis in SH-SY5Y cells.** A** An MTT assay was used to detect the viability of SH-SY-5Y cells treated with different concentrations of palmitic acid (PA) for 24 h. **B** Western blotting was performed to detect Bax and Bcl-2 expression in SH-SY-5Y cells treated with 150 µM PA for different duration. **C** Related to B, the relative quantitative analysis of Bax and Bcl-2 expression in SH-SY-5Y cells. **D** The expression levels of ERα, ERβ and GPER were detected by immunofluorescence in SH-SY-5Y cells with or without 150 µM PA treatment for 24 h. **E** Related to **D**, the quantitative analysis of the mean optical density of ERα, ERβ and GPER expression in SH-SY-5Y cells. **F** The expression levels of ERα, ERβ and GPER were detected by western blot in SH-SY-5Y cells with or without 150 µM PA treatment for 24 h. **G** Related to **F**, the relative quantitative analysis of ERα, ERβ and GPER expression in SH-SY-5Y cells. **H** The expression levels of PSD-95 and Tau were detected by immunofluorescence in SH-SY-5Y cells with or without 150 µM PA treatment for 24 h. **I** Related to **H**, the quantitative analysis of the mean optical density of PSD-95 and Tau expression in SH-SY-5Y cells. **J** The expression levels of PSD-95, Tau, Bax, and Bcl-2 were detected by western blot in SH-SY-5Y cells with or without 150 µM PA treatment for 24 h. **K** Relative quantitative analysis of PSD-95, Tau, Bax, and Bcl-2 expression in SH-SY-5Y cells. **L** An MTT assay was used to detect the viability of SH-SY-5Y cells treated with or without 150 µM PA for 24 h. **A** and **L**, n = 6. **B**–**K**, n = 3. In the indicated comparison, *P < 0.05, **P < 0.01, ***P < 0.001, ****P < 0.0001

Compared with the control group, different concentrations of palmitic acid (PA, 50 µM, 100 µM, 200 µM, 400 µM, 800 µM, or 1600 µM) induced a significant decrease in cell viability after 24 h treatment in SH-SY5Y cells, and the IC50 of PA to SH-SY5Y cells was close to 150 µM (Fig. 5A). Bax expression was increased and Bcl-2 expression was decreased after SH-SY5Y cells were treated with 150 µM PA for 24 h (Fig. 5B, C). The expression of ERα, ERβ and GPR30 in SH-SY5Y cells was decreased after treatment with 150 µM PA for 24 h compared with the control group, suggesting that the lipid overload environment created by PA inhibited the expression of ERs in nerve cells (Fig. 5D–G). Compared with the control group, after treatment with 150 µM PA for 24 h, PSD-95 expression was decreased and Tau expression was increased significantly in SH-SY5Y cells (Fig. 5H, I). Compared with the control group, the protein expression levels of PSD-95 and Bcl-2 were significantly decreased, and the protein expression levels of Tau and Bax were significantly increased in SH-SY5Y cells treated with 150 µM PA for 24 h (Fig. 5J, K). The results of the cell viability assay confirmed that the cell viability decreased by approximately 50% after SH-SY5Y cells were treated with 150 µM PA for 24 h (Fig. 5L).

### ERα, ERβ, and GPER in common mediate the protective effect of lipid-lowering agents on SH-SY5Y cells

**Fig. 6:**
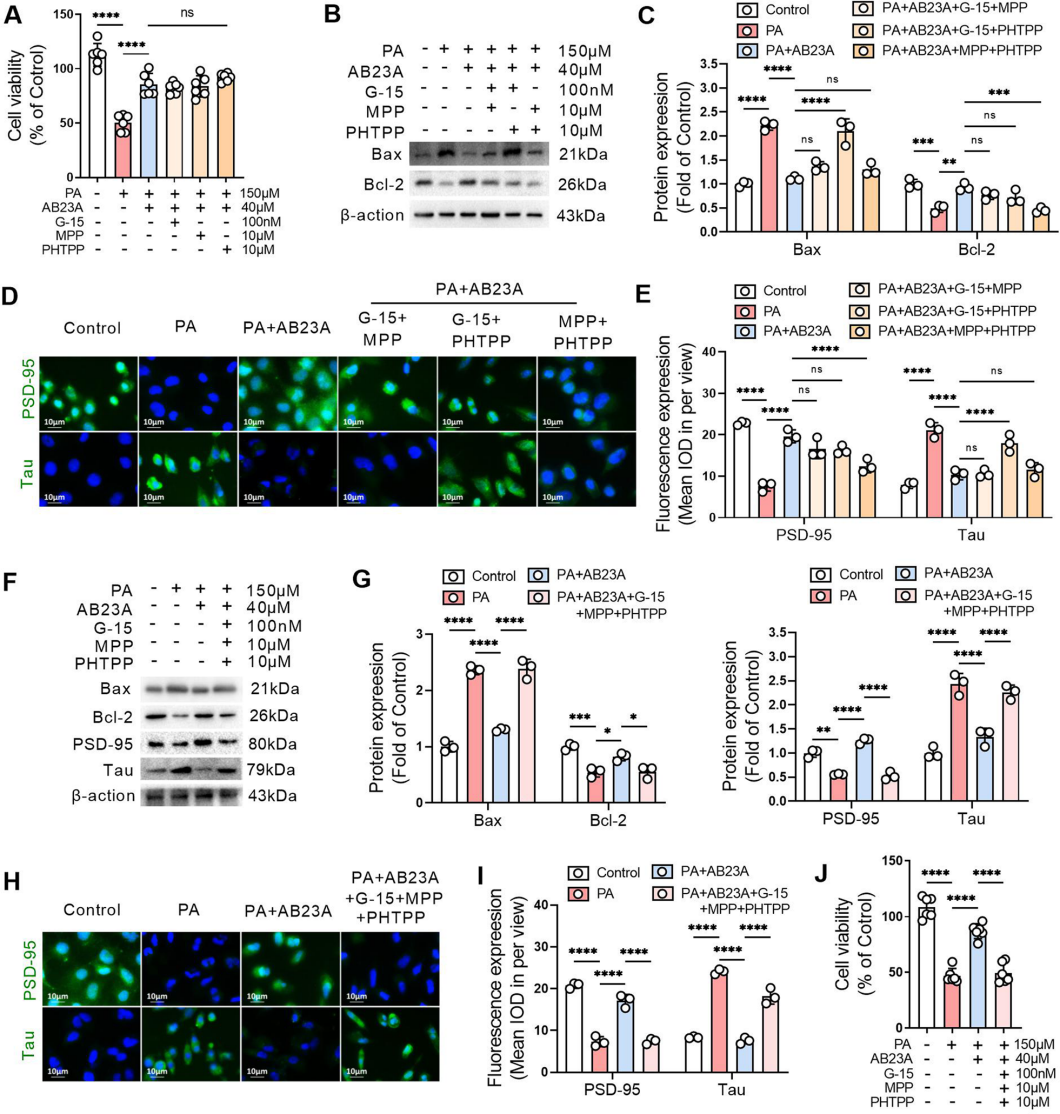
ERα, ERβ, and GPER in common mediate the protective effect of lipid-lowering agents on SH-SY5Y cells. **A** In PA-treated SH-SY-5Y cells, AB23A was used alone or in combination with G-15, MPP or PHTPP for 24 h, and cell viability was detected by MTT assay. **B** G-15, MPP, or PHTPP were paired and acted together with AB23A on PA-treated SH-SY-5Y cells for 24 h. The expression levels of Bax and Bcl-2 were detected by western blot. **C** Related to B, the relative quantitative analysis of Bax and Bcl-2 expression in SH-SY-5Y cells. **D** G-15, MPP, or PHTPP were paired and acted together with AB23A on PA-treated SH-SY-5Y cells for 24 h. The expression levels of PSD-95 and Tau were detected by immunofluorescence. **E** Related to D, the quantitative analysis of the mean optical density of PSD-95 and Tau expression in SH-SY-5Y cells. **F** SH-SY-5Y cells were treated with G-15, MPP and PHTPP combined with AB23A and treated with PA for 24 h. The expression levels of Bax, Bcl-2, PSD-95 and Tau were detected by western blotting. **G** Relative quantitative analysis of Bax, Bcl-2, PSD-95 and Tau expression in SH-SY-5Y cells. **H** SH-SY-5Y cells were treated with G-15, MPP and PHTPP combined with AB23A and treated with PA for 24 h. The expression levels of PSD-95 and Tau were detected by immunofluorescence. **I** Related to H, the quantitative analysis of the mean optical density of PSD-95 and Tau expression in SH-SY-5Y cells. **J** SH-SY-5Y cells incubated with G-15, MPP and PHTPP in combination with AB23A and treated with PA for 24 h. Cell viability was detected by MTT assay. **A** and **J**, n = 6. **B**–**I**, n = 3. In the indicated comparison, *P < 0.05, **P < 0.01, ***P < 0.001, ****P < 0.0001

Alisol B 23-acetate (AB23A, 40 µM), a widely reported lipid-lowering agent, significantly increased the viability of SH-SY5Y cells cultured with 150 µM PA for 24 h, and this effect of AB23A was not blocked by the GPER inhibitor G-15, the ERα inhibitor MPP, or the ERβ inhibitor PHTPP (Fig. 6A). AB23A significantly reduced the protein expression level of Bax and increased the protein expression level of Bcl-2 in PA-treated SH-SY5Y cells. When G-15, MPP or PHTPP were paired and acted together with AB23A on SH-SY5Y cells, two pairs of them did not affect the inhibitory effect on Bax protein expression or the upregulation effect on Bcl-2 protein expression of AB23A (Fig. 6B, C). AB23A significantly increased PSD-95 expression and decreased Tau expression in PA-treated SH-SY5Y cells. Moreover, when G-15, MPP or PHTPP were paired and together with AB23A on SH-SY5Y cells, two pairs of them did not affect the inhibitory effect on Tau expression or the upregulation effect on PSD-95 expression of AB23A (Fig. 6D, E). When G-15, MPP and PHTPP were all combined with AB23A and used in PA-treated SH-SY5Y cells, the effect of AB23A on increasing the protein expression levels of PSD-95 and Bcl-2 was abolished, and the effect of AB23A on decreasing the protein expression levels of Tau and Bax was prohibited (Fig. 6F–I). The MTT results confirmed that AB23A increased the viability of SH-SY5Y cells incubated with PA, which was inhibited by the combined application of G-15, MPP and PHTPP (Fig. 6J).

### Activating ERs improves hippocampal damage and cognitive impairment caused by ovariectomy in LDLR^−/−^ mice

**Fig. 7:**
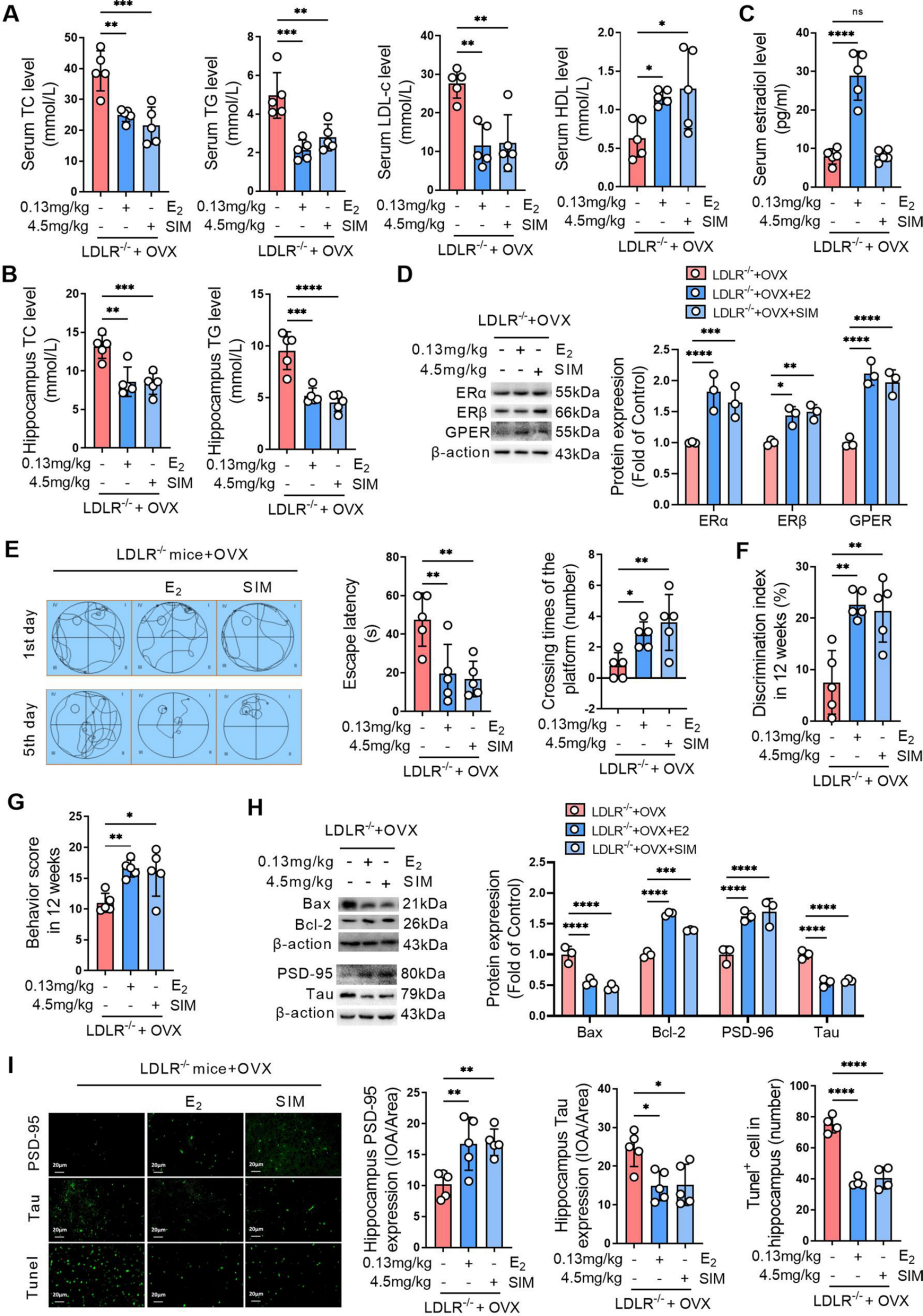
Activating ERs improves hippocampal damage and cognitive impairment caused by ovariectomy in LDLR^−/−^ mice. Bilateral ovariectomy was performed in LDLR^−/−^ mice fed a HFD to simulate a postmenopausal stage. The mice were treated with 0.13 mg/kg estradiol (E2) or 4.5 mg/kg simvastatin (SIM) for 90 days. **A** Serum of mice was obtained after 90 days of treatment, and the levels of TC, TG, LDL-c and HDL-c were detected using biochemical kits. **B** The levels of TC and TG in hippocampal homogenates were detected using biochemical kits. **C** The serum estradiol levels were detected using an ELISA kit. **D** The expression of ERα, ERβ and GPER in the hippocampal tissues of mice was detected by western blot, and the relative quantitative analysis of ERα, ERβ and GPER expression was performed. **E** The learning and memory ability of mice was evaluated by the Morris water maze after 90 days of treatment. The left panel shows the swimming paths of mice on the first and fifth training days of the Morris water maze test. The middle panel indicates the escape latency of mice on the fifth training day of the Morris water maze test. The right panel indicates the number of platform crossings on the sixth day of the Morris water maze test. **F** The learning and memory abilities of mice were evaluated by the novel object recognition test after 90 days of treatment. **G** Spatial memory of mice was evaluated by the Y-maze task after 90 days of treatment. **H** The expression of Bax, Bcl-2, PSD-95, and Tau in the hippocampal tissues of mice was detected by western blot, and the relative quantitative analysis of Bax, Bcl-2, PSD-95, and Tau expression was performed. **I** The expression of PSD-95 and Tau was detected by immunofluorescence staining, and the cell apoptosis was detected by tunel staining in the hippocampal tissues of mice after 90 days of administration. The magnification and scale bar are marked in the representative images. The expression levels of PSD-95 and Tau in the hippocampus were quantified by the mean optical density, and the number of apoptotic cells in each view was counted. **A**, **B** and **E**–**G**, n = 5. **D** and **H**, n = 3. I, n = 4–5. In the indicated comparison, *P < 0.05, **P < 0.01, ***P < 0.001, ****P < 0.0001

Serum TC, TG, and LDL-C levels, as well as TC and TG levels in the hippocampus, were significantly decreased and HDL-C levels were significantly increased in ovariectomized LDLR^−/−^ mice treated with estradiol (E2) or simvastatin (SIM) (Fig. 7A, B). These results indicated that both E2 and SIM could significantly improve lipid disorders in ovariectomized mice. E2 significantly increased serum estradiol levels in ovariectomized LDLR^−/−^ mice, but SIM did not affect serum estradiol levels in ovariectomized LDLR^−/−^ mice (Fig. 7C). E2 and SIM significantly increased the ERα, ERβ and GPER expression levels in hippocampal tissues of ovariectomized LDLR^−/−^ mice (Fig. 7D). The Morris water maze showed that 90 days of E2 or SIM treatment decreased escape latency and increased the number of platform crossings in ovariectomized LDLR^−/−^ mice (Fig. 7E). New object recognition test results showed that 90 days of E2 or SIM treatment increased the discrimination index in ovariectomized LDLR^−/−^ mice (Fig. 7F). The Y maze task showed that 90 days of E2 or SIM treatment significantly increased the alternative behavior score in ovariectomized LDLR^−/−^ mice (Fig. 7G). After 90 days of E2 or simvastatin treatment, the expression levels of PSD-95 and Bcl-2 were significantly increased, while Tau and Bax were significantly decreased in the hippocampus of ovariectomized LDLR^−/−^ mice (Fig. 7H). After 90 days of treatment with E2 or SIM, immunofluorescence staining revealed pronounced alterations in the expression of PSD-95 and Tau in the hippocampal tissues of ovariectomized LDLR−/− mice. Specifically, increased PSD-95 and reduced Tau expressions were observed (Fig. 7I). Furthermore, TUNEL staining underscored a notable reduction in apoptosis within the hippocampal regions of mice treated with E2 or SIM. The quantification reaffirmed these findings, showcasing elevated PSD-95 and diminished Tau levels, along with a significant drop in apoptotic cells in the treated groups compared to ovariectomized LDLR^−/−^ mice (Fig. 7I).

## Discussion

Estrogen can prevent cognitive dysfunction by adjusting neurotransmitters, increasing cerebral blood flow, regulating growth proteins related to axon elongation, and weakening neurotoxicity [19]. An epidemiological survey showed that female life expectancy has increased from 50 years to 83 years, and the average age of spontaneous menopause has remained stable at 50–51 years [20]. As a result, women have a prolonged perimenopausal period with low estrogen levels, which increases the risk of cognitive impairment. The loss of estrogen and its receptor functions exacerbates metabolic disorders in menopause [6], and it is well established that abnormal lipid metabolism is an independent risk factor for cognitive impairment [7]. In the current study, we provide direct evidence that the postmenopausal state amplifies lipid dyslipidemia and exacerbates cognitive dysfunction through the ERs pathway, and that estrogen supplementation or lipid reduction is an effective way to ameliorate postmenopausal dyslipidemia, hippocampal damage, and cognitive dysfunction.

First, we analyzed two data sets (GSE36318 and GSE1297) from the GEO database and showed that more than 40% of the differentially expressed genes in normal breast aspirates from postmenopausal women were enriched in cellular lipid metabolic processes. Compared with women with incipient cognitive impairment, the differentially expressed genes in the hippocampus of women with severe cognitive impairment were enriched in the estrogen signaling pathway and lipid metabolic process. Analysis of these data suggests that abnormal estrogen signaling and lipid disorders in the postmenopausal stage are potential drivers of cognitive dysfunction in older women.

The biggest problem faced by patients with cognitive impairment is the loss of learning and memory ability, which involves learning ability, short-term and long-term memory, mental and emotional, personal social behavior and many other aspects [21]. We found that, for C57BL/6J mice, a high-fat diet alone did not lead to cognitive impairment. LDLR knockout in mice leads to hyperlipidemia and the same does not result in cognitive impairment. When a high-fat diet was given to LDLR^−/−^ mice, more severe hyperlipidemia was present and mild cognitive impairment was generated. When LDLR^−/−^ mice fed a high-fat diet underwent bilateral ovariectomy, the lipid levels in the blood, liver, and hippocampus were significantly increased, and cognitive function was seriously damaged. These findings suggest that dyslipidemia and postmenopausal state are risk factors for the development of cognitive impairment and that postmenopausal status is an independent promoter of dyslipidemia and cognitive impairment.

Many studies have proven that cognitive impairment is related to various factors, and changes in neurotransmitters are the most important [22]. For decades, studies on the effects of neurotransmitters on cognitive impairment have made continuous progress, and studies on combating cognitive impairment by regulating neurotransmitters have increased in recent years [23]. Regulating the levels of neurotransmitters in the brain, including ACh, DA and NE, can alleviate cognitive dysfunction [24]. Most of the drugs approved by the FDA for the treatment of AD are inhibitors of AchE, and their mechanism is to inhibit the activity of AchE, thereby increasing the content of ACh neurotransmitters in the brain [25]. Our present results showed that DA and NE levels were significantly decreased in the hippocampal tissues of mice after high-fat diet, LDLR knockout and ovariectomy. The activity of AchE increased and AChT decreased in LDLR^−/−^ mice fed a high-fat diet after ovariectomy, which may be responsible for the remarkably reduced Ach content in ovariectomized LDLR^−/−^ mice.

In addition to neurotransmitters, the loss of synaptic plasticity and integrity is another important cause of cognitive dysfunction [26]. Changes in the content and distribution of Nissl bodies are important indicators of neuronal function injury. Normal central regions are rich in Nissl bodies, but when neurons are damaged, the content of Nissl bodies decreases [27]. Current results show that the content and distribution of Nissl bodies in the hippocampus of LDLR^−/−^ mice fed a high-fat diet are significantly decreased, which cooccurred with synaptic damage and hippocampal neuron apoptosis. All of these phenomena were further exacerbated by ovariectomy in LDLR^−/−^ mice. Postsynaptic denser (PSD), as a complex of signaling molecules in the postsynaptic membrane after excitation, is an important substance for synaptic transmission function [28]. As the highest content of microtubule-related protein, Tau protein content and phosphorylated Tau protein content in the brain of AD patients increase significantly, and abnormal phosphorylation of Tau promotes synaptic loss and neuron damage [29]. Our results confirm that PSD-95 expression in the hippocampus was significantly reduced, while Tau protein expression was significantly increased in mice in the postmenopausal stage, which may be the reason hippocampal damage and cognitive dysfunction occurred in ovariectomized mice. As we observed, with the change in the expression of PSD-95 and Tau, the increase in Bax expression and a decrease in Bcl-2 expression emerged in hippocampal ovariectomized mice. A range of postmenopausal effects are closely related to reduced estrogen levels and decreased estrogen receptor function, including cognitive dysfunction [30].

In the current study, ovariectomized mice showed significant reductions in estradiol levels and ERα, ERβ, and GPER expression, and they showed significant correlations with dyslipidemia and cognitive dysfunction. ERα and ERβ regulate gene transcription by binding to specific sequences in the promoters of target genes, which is a classic gene transcription effect [31]. GPER mainly activates intracellular second messengers, regulates cAMP, and activates protein kinases through signal transduction pathways, thus leading to indirect changes in gene expression [32]. To investigate the relationship between ERα, ERβ and GPER and dyslipidemia in nerve cells, we incubated SH-SY5Y cells with palmitic acid (PA) to replicate a nerve damage cell model. ERα, ERβ and GPER are expressed positively in SH-SY5Y cells [33], so SH-SY5Y cells are often used as an in vitro cell model to research the protective effect of estrogen receptors on neurons [34, 35]. However, PA can trigger neuronal apoptosis and neuroinflammation, leading to cognitive impairment [36]. Our findings are consistent with previous reports that neuronal apoptosis increases with the application of PA [37]. Moreover, PA resulted in a decrease in PSD-95 expression and an increase in Tau expression in SH-SY5Y cells. Interestingly, PA led to decreased ERα, ERβ and GPER expression in SH-SY5Y cells, which is inconsistent with our in vivo results. In vivo, only ovariectomy leads to decreased estradiol levels and ERs expression, and hyperlipidemia alone, including LDLR knockout or a high-fat diet, does not affect estradiol levels and ERs expression in mice. The in vivo results may indicate a systemic regulatory effect of the hormone system.

In vitro, we demonstrated that ERα, ERβ and GPER jointly mediate the protective effects of lipid-lowering agents on nerve cells. Specifically, AB23A, a substance that can significantly reduce blood lipids and lipid accumulation in the liver and intestines [38–40], increases PSD-95 and Bcl-2 expression and decreases Tau and Bax expression in SH-SY5Y cells. The paired application of GPER inhibitor G15, ER α inhibitor MPP and ER β inhibitor PHTPP did not affect the protective effect of AB23A on SH-SY5Y cells. However, the combined application of G-15, MPP, and PHTPP reversed the inhibitory effect of AB23A on SH-SY5Y cell apoptosis. These results highlight the important role of ERs in the use of reagents to improve the activity and function of nerve cells.

Previous studies have shown that long-term treatment with estradiol improves cognitive impairment and restores synaptic plasticity in ovariectomized rhesus monkeys [41]. Simvastatin has also been proven to reduce obesity-induced cognitive dysfunction in rats [42]. So, we next treated bilateral ovariectomized LDLR^−/−^ mice with estradiol or simvastatin to evaluate their effects on cognitive function. Our results showed that in ovariectomized mice, estradiol increased serum estradiol levels and ERs expression, reduced hyperlipidemia, and decreased apoptosis of nerve cells in the hippocampus to improve cognitive function. Simvastatin reduced hyperlipidemia and decreased apoptosis of nerve cells in the hippocampus to improve cognitive function, without affecting serum estradiol levels, but upregulating ERs expression. These findings highlight the central role of hyperlipidemia in promoting cognitive impairment in the postmenopausal stage and suggest that regulating estrogen and its receptor function is an effective way to ameliorate cognitive impairment in the postmenopausal stage.

In conclusion, we confirmed that postmenopausal women and women with cognitive impairment have abnormal lipid-related processes and signals. Decreased estradiol levels and ERs expression in the postmenopausal period contribute to lipid disorders and cognitive dysfunction. Moreover, we demonstrated that ERα, ERβ and GPER jointly mediate the protective effect of lipid-lowering agents on nerve cells, and supplementing estradiol or lowering lipids is an effective way to improve hippocampal damage and cognitive dysfunction caused by hyperlipidemia in the postmenopausal period by up-regulating ERs.

## Data Availability

The datasets used and/or analyzed during the current study are available from the corresponding author on reasonable request.

## References

[1] McNeil MA, Merriam SB (2021). Menopause. Ann Intern Med.

[2] Hara Y, Waters EM, McEwen BS, Morrison JH (2015). Estrogen effects on cognitive and synaptic health over the lifecourse. Physiol Rev.

[3] Hu J, Chu K, Song Y, Chatooah ND, Ying Q, Ma L, Zhou J, Qu F, Zhou J (2017). Higher level of circulating estradiol is associated with lower frequency of cognitive impairment in Southeast China. Gynecol Endocrinol.

[4] Mosconi L, Berti V, Quinn C, McHugh P, Petrongolo G, Varsavsky I, Osorio RS, Pupi A, Vallabhajosula S, Isaacson RS (2017). Sex differences in Alzheimer risk: brain imaging of endocrine vs chronologic aging. Neurology.

[5] Santoro N, Epperson CN, Mathews SB (2015). Menopausal symptoms and their management. Endocrinol Metab Clin North Am.

[6] Mezzullo M, Gambineri A, Di Dalmazi G, Fazzini A, Magagnoli M, Baccini M, Vicennati V, Pelusi C, Pagotto U, Fanelli F (2021). Steroid reference intervals in women: influence of menopause, age and metabolism. Eur J Endocrinol.

[7] O’Brien PD, Hinder LM, Callaghan BC, Feldman EL (2017). Neurological consequences of obesity. Lancet Neurol.

[8] Lin YS, Liu CK, Lee HC, Chou MC, Ke LY, Chen CH, Chen SL (2021). Electronegative very-low-density lipoprotein induces brain inflammation and cognitive dysfunction in mice. Sci Rep.

[9] Bogie JFJ, Haidar M, Kooij G, Hendriks JJA (2020). Fatty acid metabolism in the progression and resolution of CNS disorders. Adv Drug Deliv Rev.

[10] Huang W, Li Z, Zhao L, Zhao W (2017). Simvastatin ameliorate memory deficits and inflammation in clinical and mouse model of Alzheimer’s Disease via modulating the expression of miR-106b. Biomed Pharmacother.

[11] Pratchayasakul W, Sa-Nguanmoo P, Sivasinprasasn S, Pintana H, Tawinvisan R, Sripetchwandee J, Kumfu S, Chattipakorn N, Chattipakorn SC (2015). Obesity accelerates cognitive decline by aggravating mitochondrial dysfunction, insulin resistance and synaptic dysfunction under estrogen-deprived conditions. Horm Behav.

[12] Meng Q, Ma M, Zhang W, Bi Y, Cheng P, Yu X, Fu Y, Chao Y, Ji T, Li J (2021). The gut microbiota during the progression of atherosclerosis in the perimenopausal period shows specific compositional changes and significant correlations with circulating lipid metabolites. Gut Microbes.

[13] Meng Q, Li Y, Ji T, Chao Y, Li J, Fu Y, Wang S, Chen Q, Chen W, Huang F (2021). Estrogen prevent atherosclerosis by attenuating endothelial cell pyroptosis via activation of estrogen receptor alpha-mediated autophagy. J Adv Res.

[14] Meng Q, Li J, Chao Y, Bi Y, Zhang W, Zhang Y, Ji T, Fu Y, Chen Q, Zhang Q (2020). : beta-estradiol adjusts intestinal function via ERbeta and GPR30 mediated PI3K/AKT signaling activation to alleviate postmenopausal dyslipidemia. Biochem Pharmacol.

[15] Meng Q, Yu X, Chen Q, Wu X, Kong X, Wang S, Cai D, Cheng P, Li Y, Bian H (2020). Liuwei Dihuang soft capsules inhibits the phenotypic conversion of VSMC to prevent the menopausal atherosclerosis by up-regulating the expression of myocardin. J Ethnopharmacol.

[16] Russell JK, Jones CK, Newhouse PA (2019). The role of estrogen in brain and cognitive aging. Neurotherapeutics.

[17] Tang SS, Ren Y, Ren XQ, Cao JR, Hong H, Ji H, Hu QH (2019). ERalpha and/or ERbeta activation ameliorates cognitive impairment, neurogenesis and apoptosis in type 2 Diabetes Mellitus mice. Exp Neurol.

[18] Tang SS, Ren Y, Xu LJ, Cao JR, Hong H, Ji H, Hu QH (2018). Activation of ERalpha and/or ERbeta ameliorates cognitive impairment and apoptosis in streptozotocin-induced diabetic mice. Horm Behav.

[19] Brinton RD, Yao J, Yin F, Mack WJ, Cadenas E (2015). Perimenopause as a neurological transition state. Nat Rev Endocrinol.

[20] Pompili A, Arnone B, Gasbarri A (2012). Estrogens and memory in physiological and neuropathological conditions. Psychoneuroendocrinology.

[21] McWhirter L, Ritchie C, Stone J, Carson A (2020). Functional cognitive disorders: a systematic review. Lancet Psychiatry.

[22] Gauthier S, Reisberg B, Zaudig M, Petersen RC, Ritchie K, Broich K, Belleville S, Brodaty H, Bennett D, Chertkow H (2006). Mild cognitive impairment. Lancet.

[23] Holland N, Robbins TW, Rowe JB (2021). The role of noradrenaline in cognition and cognitive disorders. Brain.

[24] Kandimalla R, Reddy PH (2017). Therapeutics of neurotransmitters in Alzheimer’s Disease. J Alzheimers Dis.

[25] Lombardo S, Maskos U (2015). Role of the nicotinic acetylcholine receptor in Alzheimer’s Disease pathology and treatment. Neuropharmacology.

[26] Hong S, Beja-Glasser VF, Nfonoyim BM, Frouin A, Li S, Ramakrishnan S, Merry KM, Shi Q, Rosenthal A, Barres BA (2016). Complement and microglia mediate early synapse loss in Alzheimer mouse models. Science.

[27] Xie Y, Yan B, Hou M, Zhou M, Liu C, Sun M, He K, Fang C, Chen Y, Huang L (2021). Erzhi pills ameliorate cognitive dysfunction and alter proteomic hippocampus profiles induced by d-galactose and Abeta1-40 injection in ovariectomized Alzheimer’s Disease model rats. Pharm Biol.

[28] Zeng M, Chen X, Guan D, Xu J, Wu H, Tong P, Zhang M (2018). Reconstituted postsynaptic density as a molecular platform for understanding synapse formation and plasticity. Cell.

[29] Bassil F, Brown HJ, Pattabhiraman S, Iwasyk JE, Maghames CM, Meymand ES, Cox TO, Riddle DM, Zhang B, Trojanowski JQ (2020). Amyloid-Beta (abeta) plaques promote seeding and spreading of alpha-synuclein and tau in a mouse model of Lewy Body disorders with Abeta Pathology. Neuron.

[30] Gleason CE, Dowling NM, Wharton W, Manson JE, Miller VM, Atwood CS, Brinton EA, Cedars MI, Lobo RA, Merriam GR (2015). Effects of hormone therapy on cognition and mood in recently postmenopausal women: findings from the randomized, controlled KEEPS-cognitive and affective study. PLoS Med.

[31] Pakdel F (2018). Molecular pathways of estrogen receptor action. Int J Mol Sci.

[32] Olde B, Leeb-Lundberg LM (2009). GPR30/GPER1: searching for a role in estrogen physiology. Trends Endocrinol Metab.

[33] Ding X, Gao T, Gao P, Meng Y, Zheng Y, Dong L, Luo P, Zhang G, Shi X, Rong W (2019). Activation of the G protein-coupled estrogen receptor elicits store calcium release and phosphorylation of the mu-opioid receptors in the human neuroblastoma SH-SY5Y cells. Front Neurosci.

[34] Santos CC, Munoz P, Almeida A, de Lima David JP, David JM, Lima Costa S, Segura-Aguilar J, Silva VDA (2020). The flavonoid agathisflavone from *Poincianella pyramidalis* prevents aminochrome neurotoxicity. Neurotox Res.

[35] Tsai MC, Lin SH, Hidayah K, Lin CI (2019). Equol pretreatment protection of SH-SY5Y cells against abeta (25-35)-induced cytotoxicity and cell-cycle reentry via sustaining estrogen receptor alpha expression. Nutrients.

[36] Jo D, Yoon G, Song J (2021). Role of exendin-4 in brain insulin resistance, mitochondrial function, and neurite outgrowth in neurons under palmitic acid-induced oxidative stress. Antioxid (Basel).

[37] Ng YW, Say YH (2018). Palmitic acid induces neurotoxicity and gliatoxicity in SH-SY5Y human neuroblastoma and T98G human glioblastoma cells. PeerJ.

[38] Meng Q, Duan XP, Wang CY, Liu ZH, Sun PY, Huo XK, Sun HJ, Peng JY, Liu KX (2017). Alisol B 23-acetate protects against non-alcoholic steatohepatitis in mice via farnesoid X receptor activation. Acta Pharmacol Sin.

[39] Chen Q, Chao Y, Zhang W, Zhang Y, Bi Y, Fu Y, Cai D, Meng Q, Li Y, Bian H (2020). Activation of estrogen receptor alpha (ERalpha) is required for Alisol B23-acetate to prevent post-menopausal atherosclerosis and reduced lipid accumulation. Life Sci.

[40] Yu XC, Fu Y, Bi YH, Zhang WW, Li J, Ji T, Chao Y, Meng QH, Chen Q, Ma MH (2020). Alisol B 23-acetate activates ABCG5/G8 in the Jejunum via the LXRalpha/ACAT2 pathway to relieve Atherosclerosis in ovariectomized ApoE(-/-) mice. Aging.

[41] Crimins JL, Wang AC, Yuk F, Puri R, Janssen WGM, Hara Y, Rapp PR, Morrison JH (2017). Diverse synaptic distributions of G protein-coupled estrogen receptor 1 in monkey prefrontal cortex with aging and menopause. Cereb Cortex.

[42] Lorenzoni R, Davies S, Cordenonsi LM, Vicosa J, Mezzomo NJ, de Oliveira AL, Carmo GMD, Raffin RP, Alves OL, Vaucher RA (2020). Lipid-core nanocapsules containing simvastatin improve the cognitive impairment induced by obesity and hypercholesterolemia in adult rats. Eur J Pharm Sci.

